# Calcifediol (25-hydroxyvitamin D) improvement and calcium-phosphate metabolism of alendronate sodium/vitamin D_3_ combination in Chinese women with postmenopausal osteoporosis: a post hoc efficacy analysis and safety reappraisal

**DOI:** 10.1186/s12891-018-2090-y

**Published:** 2018-07-03

**Authors:** Er-Yuan Liao, Zhen-Lin Zhang, Wei-Bo Xia, Hua Lin, Qun Cheng, Li Wang, Yong-Qiang Hao, De-Cai Chen, Hai Tang, Yong-De Peng, Li You, Liang He, Zhao-Heng Hu, Chun-Li Song, Fang Wei, Jue Wang, Lei Zhang

**Affiliations:** 1 The Second Xiangya Hospital, Central South University, Changsha, China; 2 The Sixth People’s Hospital, Shanghai Jiaotong University, Shanghai, China; 3 Department of Endocrinology, Key Laboratory of Endocrinology, Ministry of Health, Peking Union Medical College Hospital, Chinese Academy of Medical Sciences, Shuaifuyuan No. 1, Wangfujing, Dongcheng District, Beijing, 100730 China; 4 Nanjing Drum Tower Hospital, Nanjing, China; 5 Huadong Hospital Affiliated to Fudan University, Shanghai, China; 6 Tianjin Hospital, Tianjin, China; 7 The Ninth People’s Hospital, Shanghai, China; 8 West China Hospital, West China School of Medicine, Sichuan University, Chengdu, China; 9 Beijing Friendship Hospital, Capital Medical University, Beijing, China; 10 The First People’s Hospital, Shanghai, China; 11 Beijing Jishuitan Hospital, Beijing, China; 12 Peking University People’s Hospital, Beijing, China; 13 Peking University Third Hospital, Beijing, China; 14 Global Medical Affairs, Merck Sharp & Dohme China, Shanghai, China

**Keywords:** Postmenopausal osteoporosis, Alendronate sodium, Calcitriol, Calcifediol, Calcium/phosphate metabolism

## Abstract

**Background:**

Vitamin D (VD) insufficiency or deficiency is a frequent comorbidity in Chinese women with postmenopausal osteoporosis (PMO). The present study aimed to investigate 25-hydroxyvitamin D [25(OH) D] improvement and calcium-phosphate metabolism in Chinese PMO patients treated with 70 mg of alendronate sodium and 5600 IU of vitamin D_3_ (ALN/D5600).

**Methods:**

Chinese PMO women (*n* = 219) were treated with 12-month ALN/D5600 (*n* = 111) or calcitriol (*n* = 108). Changes in 25(OH) D at month 12 were post hoc analyzed by the baseline 25 (OH) D status using the longitudinal analysis. The main safety outcome measures included serum calcium and phosphate and 24-h urine calcium, and the repeated measures mixed model was used to assess the frequencies of the calcium-phosphate metabolic disorders.

**Results:**

Absolute change in mean serum 25(OH) D level was the greatest in VD-deficient patients and least in VD-sufficient patients at months six and 12 (both, *P* < 0.01). Serum calcium level remained significantly lower in the ALN/D5600 treatment group than in the calcitriol treatment group throughout the 12 months. Mean 24-h urine calcium slightly increased in the ALN/D5600 treatment group and significantly increased in the calcitriol treatment group (+ 1.1 and + 0.9 mmol/L at months six and 12; both, *P* < 0.05). Calcitriol treatment was associated with more frequent hypercalciuria at month six (9.4% vs. 18.5%, *P* = 0.05), but not at month 12 (12.3% vs. 13.0%).

**Conclusion:**

Baseline VD status predicted 25(OH) D improvement in PMO patients on 12-month ALN/D5600 treatment. The daily use of 0.25 μg of calcitriol was associated with more frequent hypercalciuria at month six, compared to ALN/5600 treatment, necessitating the safety re-evaluation of calcitriol at a higher dosage.

**Electronic supplementary material:**

The online version of this article (10.1186/s12891-018-2090-y) contains supplementary material, which is available to authorized users.

## Background

Postmenopausal osteoporosis (PMO) is a major concern in women’s health worldwide, which has a prevalence of approximately 30% in Western countries [1] and approximately 40.1% in the Chinese population [2]. Fragility fracture occurs in at least 40% of Caucasian PMO women [3], who may require hospital admission and prolonged hospitalization. Furthermore, the continuous aging of the population resulted in the increased incidence of osteoporosis for both genders, particularly for women > 50 years old [4].

The prevention and treatment of osteoporosis includes calcium and vitamin D (VD) supplementation and antiresorptive therapies [5]. VD insufficiency or deficiency, defined as low levels of serum 25-hydroxyvitamin D [25(OH) D] (less than 30 and 20 ng/mL, respectively), is also a major health problem for postmenopausal women, which often coexists with osteoporosis [6]. However, it has been proven that the use of high-dose vitamin D_3_ does not clinically benefit postmenopausal women with respect to skeletal and muscular health, although this therapy increases calcium absorption [7]. A possible explanation is that loss of bone mass in postmenopausal women results from multiple factors such as ageing, decreased estrogen, obesity and other comorbidities [8]. However, the previous randomized, controlled study conducted by the investigators demonstrated that a weekly dose of alendronate sodium with vitamin D_3_ (ALN/D5600) was superior to 0.25 μg of calcitriol (1,25(OH)_2_D) daily, which is an active derivative of vitamin D_3_, with respect to lumbar spine bone mineral density (BMD) and bone turnover markers (BTMs) [9]. The further post hoc analysis performed by the investigators revealed that ALN/D5600 was more beneficial for patients with lower baseline serum C-terminal telopeptide (s-CTx) or greater on-treatment s-CTx decrease, in terms of lumbar spine BMD improvement, when compared with calcitriol, which is more beneficial for patients with lower baseline serum vitamin D or prior vertebral fracture [10].

There is a knowledge gap regarding the effect of combining vitamin D with alendronate on the VD status of Chinese PMO patients. This post hoc analysis aimed to determine to what extent the ALN/D5600 combination could improve the coexisting VD insufficiency/deficiency, and the association between baseline VD status and on-treatment serum 2 (OH) D increase, in PMO patients. Moreover, safety concerns on calcium-phosphate metabolism remained among Chinese PMO patients who received ALN/D5600 or active derivatives of vitamin D_3_. Therefore, the present study also specifically reexamined the calcium-phosphate metabolic profile of ALN/D5600, when compared with calcitriol, an active vitamin D_3_ preparation.

## Methods

### Study protocol and patients

The original study was a 6-month, randomized, open-label, active-comparator-controlled, parallel-group study with a 6-month extension to evaluate the efficacy and safety of weekly ALN/D5600 as a combination tablet vs. 0.25 μg of calcitriol daily in the treatment of osteoporosis in postmenopausal women in China (protocol number 264–01, clinicaltrials.gov number NCT01350934). The study protocol, design and inclusion/exclusion criteria were as previously reported [9]. Briefly, eligible patients were patients who were > 55 years of age and postmenopausal for at least one year, with no prior antiresorptive therapies, patients who had no prior hip fracture, patients who had no pronounced medical history other than osteoporosis, and patients had no uncontrolled primary/secondary hyperparathyroidism (as examined by iPTH at screening) or evidence of metabolic bone disease other than postmenopausal osteoporosis. The original study was sponsored by MSD China Holding Co., Ltd., and was approved by the independent ethics committees of 13 participating sites. A written informed consent was obtained for all patients before any screening procedure was performed.

### Intervention and analyzed outcomes

In the original study, patients were 1:1 randomized to ALN/D5600 (*n* = 111) or calcitriol (*n* = 108) for a total of 12 months via a randomization telephone central line. Study medications were Fosamax® Plus D (alendronate 70 mg/vitamin D35600 IU; manufactured by FROSST IBERICA. SA, a subsidiary of Merck; packaged by MSD, Australia) and Rocaltrol® (calcitriol 0.25 μg; manufactured by R.P. Scherer GmbH & Co. KG under Hoffman-La Roche AG; packaged by Roche, Shanghai, China). Generic calcium supplements (500 mg daily) were administered to all patients for the entire study period.

In the present study, treatment compliance was calculated using the following formula: Compliance (%) = number of days on therapy / number of days should be on therapy × 100. A day in the study was considered as an “On-Therapy” day when the patient took the required number of tablets from any of the bottles of the dispensed study medication. The “Number of Days Should be on Therapy” was the total number of days from randomization to the last scheduled day (or discontinued day) for treatment administration for each subject. Among all subjects, 10.8% (12/111) and 0.0% (0/108) had a treatment compliance of < 80% in the ALN/D5600 and calcitriol treated groups, respectively.

This *post-hoc* analysis was based on subject’s data collected from the original study, in which the analyzed outcomes were changes in serum 25(OH) D levels at the end of 12-month study treatment, serum 25(OH) D levels at each study visit defined as vitamin D insufficiency (< 30 ng/mL) or deficiency (< 20 ng/mL), and serum calcium-phosphate levels throughout the study and those defined as hypercalcemia (> 2.60 mmol/L) or hyperphosphatemia (> 1.46 mmol/L with an increase of > 20% from baseline). Moreover, urine calcium levels were analyzed as hypercalciuria (24-hurine calcium > 7.5 mmol/L with an increase of > 25% from baseline).

### Laboratory measurement

All laboratory measurements were performed in a central laboratory (Quest Diagnostics Clinical Trials, Shanghai, China). Serum 25(OH) D level was measured during the screening and at month 3, 6, 9and 12, respectively, using liquid chromatography-tandem mass spectrometry. Calcium-phosphate measurements were performed during the screening and at month 3, 6, 9 and 12, respectively. In addition 24-h urine calcium was determined at the time of randomization and at month six and 12. Intact PTH was performed at the time of screening to rule out primary or secondary hyperparathyroidism.

### Statistical analysis

This was a *post-hoc* analysis study on a previous reported RCT. No specific priori statistical hypothesis was made for the analyses. Therefore, no sample size consideration was performed. Changes in serum 25(OH) D levels were analyzed in the full analysis set (FAS), which comprised of randomized patients who received at least one dose of study treatment and one post-treatment observation, and no imputation on missing data was performed. Serum 25(OH) D levels were presented by box plotting by each study visit. The association between baseline and 12-month post-randomization serum 25(OH) D levels was analyzed using linear regression and a longitudinal data analysis (LDA) model. In the LDA, a mixed model with an unstructured covariance matrix was established to include the absolute change in 25(OH) D levels at month 12 as a response variable, and time, tertiles of baseline 25(OH) D and the interaction of time by tertiles of baseline 25(OH) D as categorical variables. Missing data in the LDA model were deemed missing at random (MAR). The Cochran-Armitage trend test was used to examine the trend of vitamin D insufficiency or deficiency overtime during the study treatment.

For the analysis of calcium-phosphate metabolism, all patients in the treated (APaT) population, which comprised of randomized patients who received at least one dose of study treatment, were included. Chi-square test (Bonferroni adjusted) was used to compare the proportion of patients who experienced safety events of special interest (hypercalcemia, hyperphosphatemia and hypercalciuria), as predefined. *P*-values for between-treatment differences in the percentage of patients with adverse events were calculated using the Miettinen and Nurminen method. An LDA mixed model with repeated measures was applied to compare within- and between-group differences in calcium and phosphate levels throughout the study. This model assumed different levels of calcium-phosphate from baseline for each treatment at each of the repeated time points in the analysis. Time was treated as a categorical variable so that no restriction was imposed on the trajectory of means over time. An unstructured covariance matrix was used to model the correlation among repeated measurements. *P*-values for within- and between-group comparisons were calculated using the LDA mixed model repeated measures. The absolute difference and 95% confidence interval (95% CI) of calcium and phosphate levels between the two study treatment groups were also provided.

Other possible continuous data were expressed as mean and standard deviation, and categorical data were presented as *n* (%).All statistical analyses were performed using Statistical Analysis System 9.4 (SAS Institute, Cary, North Carolina, USA) and NCSS 10 (NCSS, LLC, Kaysville, Utah, USA). A two-sided *P*-value < 0.05 was considered statistically significant, unless otherwise specified.

## Results

### VD status improvement

**Fig. 1:**
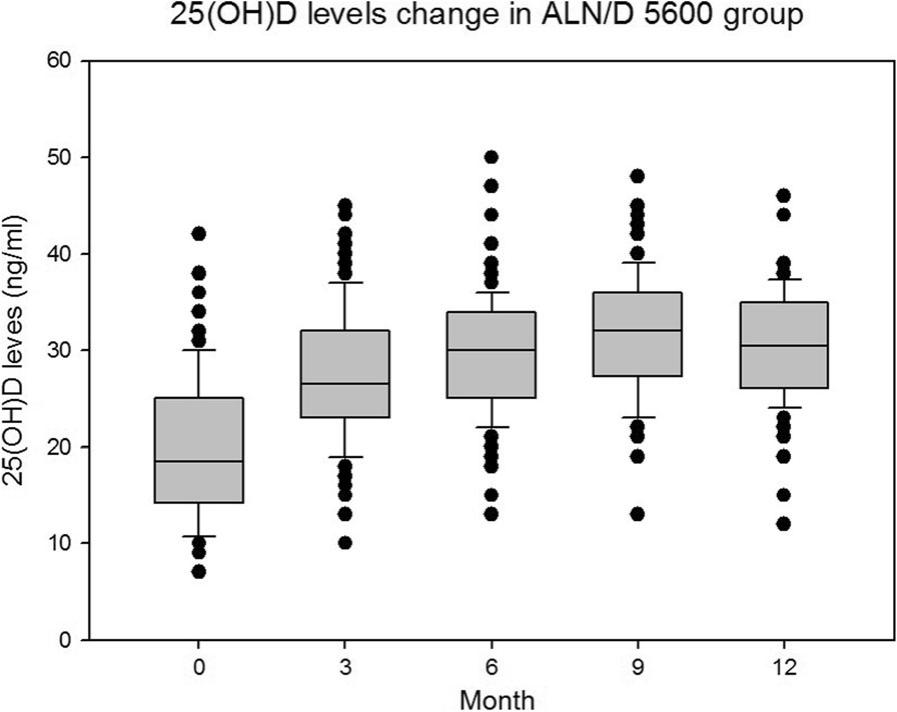
The box plot of serum 25(OH) D levels (ng/ml) in PMO patients (*n* = 96) who received ALN/D5600 treatment throughout the 12-month study period. The central thick lines represent the medians, the boxes represent the 25 and 75% quartile range, the whiskers (error bars) represent the 10th and 90th percentiles, and the black dots represent the outliers

In the ALN/D5600 treatment group, 96 patients had 12-month serum 25(OHD) data available for evaluation. The mean serum 25(OH) D level significantly increased in the ALN/D5600 treatment group at 6 months from baseline. This remained unchanged at 12 months (mean, 30.2 ng/ml; 95% CI: 18.7, 41.8 ng/ml; range, 12–46 ng/ml), and fell below the safety threshold (50 ng/ml) in all patients throughout the study period (Fig. 1). At baseline, 89.6% of patients had VD insufficiency and 54.2% of patients had VD deficiency, both of which had a significant decrease throughout the 12-month study period (*P-*values for trend < 0.001, Table 1). The proportion of VD-deficient patients decreased more dramatically than that of VD-insufficient patients. It is noteworthy that four patients had persistent VD deficiency, even though they were given 12 months of VD supplementation, and three of these patients had very low baseline serum 25(OH) D levels (Additional file 1: Table S1).

**Table 1:** VD status of PMO patients who received ALN/D5600 combination treatment throughout the 12-month study period (*n* = 96)

Percent^a^	Baseline	3 months	6 months	9 months	12 months
VD insufficiency	89.6	65.6	47.9	33.4	43.8
VD deficiency	54.2	11.5	5.2	3.1	4.2

^a^Vitamin D insufficiency was defined as serum 25(OH) D < 30 ng/mL; vitamin D deficiency was defined as serum 25(OH) D < 20 ng/mL; Test for trend *P-*values < 0.001 for VD insufficiency and deficiency using the Cochran-Armitage trend test

### Baseline VD status correlates with the 12-month 25(OH) D improvement

**Fig. 2:**
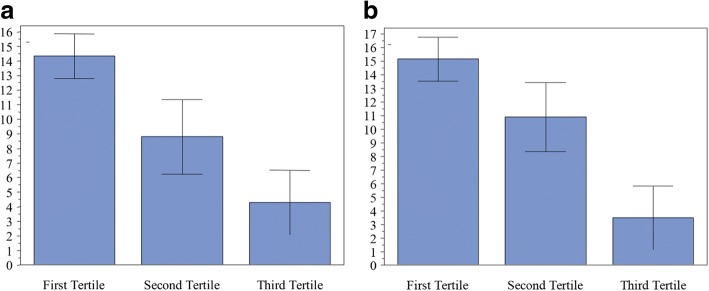
Correlation of absolute changes (ng/mL) in serum 25(OH) levels at months 6 (**a**) and 12 (**b**) with baseline 25(OH) tertiles for patients who received the 12-month ALN/D5600 treatment. A longitudinal data analysis with an unstructured covariance matrix was used to model the correlation among repeated measurements. The model includes the absolute change in 25(OH) D levels as a response variable, and includes terms for time, tertiles of baseline 25(OH) D, and the interaction of time by tertiles of baseline 25(OH) D. Error bars represent the standard deviation

**Fig. 3:**
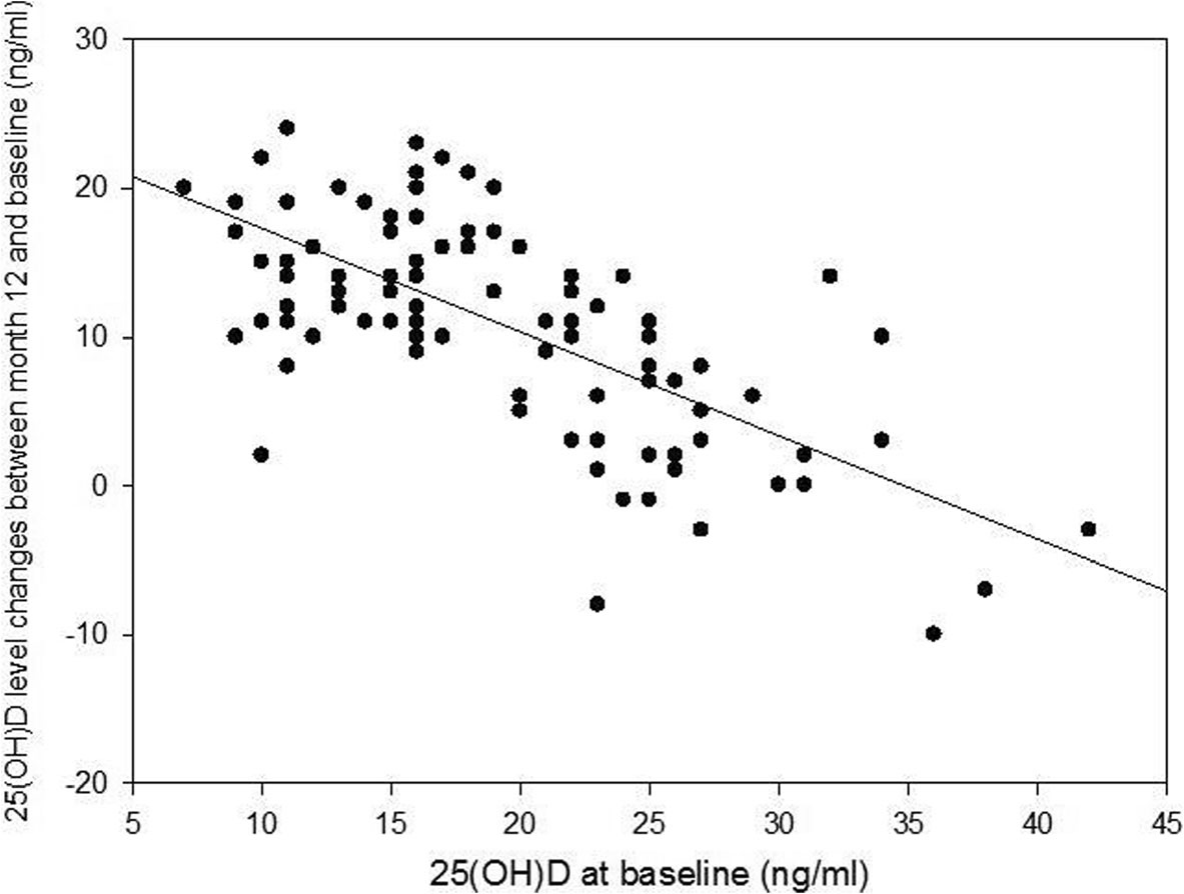
A dot plot of absolute changes in serum 25(OH) D levels (ng/mL) at year one against baseline level (ng/mL). Person correlation coefficient = − 0.70 (95% CI, [− 0.54, − 0.85]; *P* < 0.001)

Figure 2 illustrates the correlation of the absolute changes in serum 25(OH) D levels at six and 12 months with baseline VD status (the 1st tertile, VD deficiency; the 2nd tertile, VD insufficiency; the 3rd tertile, VD sufficiency). All these three tertiles had significant increases in mean serum 25(OH) D level at months six and 12 from baseline (data not shown, *P* < 0.05). Absolute change in mean serum 25(OH) D level was greatest in VD-deficient patients and least in VD-sufficient patients at months six (Fig. 2a) and 12 (Fig. 2b) (*P* < 0.01). Linear regression modeling revealed that the 1-year absolute change in serum 25(OH) D level had a significant negative correlation with baseline VD levels (Person coefficient [95% CI], − 0.70 [− 0.54, − 0.85]; *P* < 0.001; Fig. 3).

### Calcium-phosphate metabolic profile

**Fig. 4:**
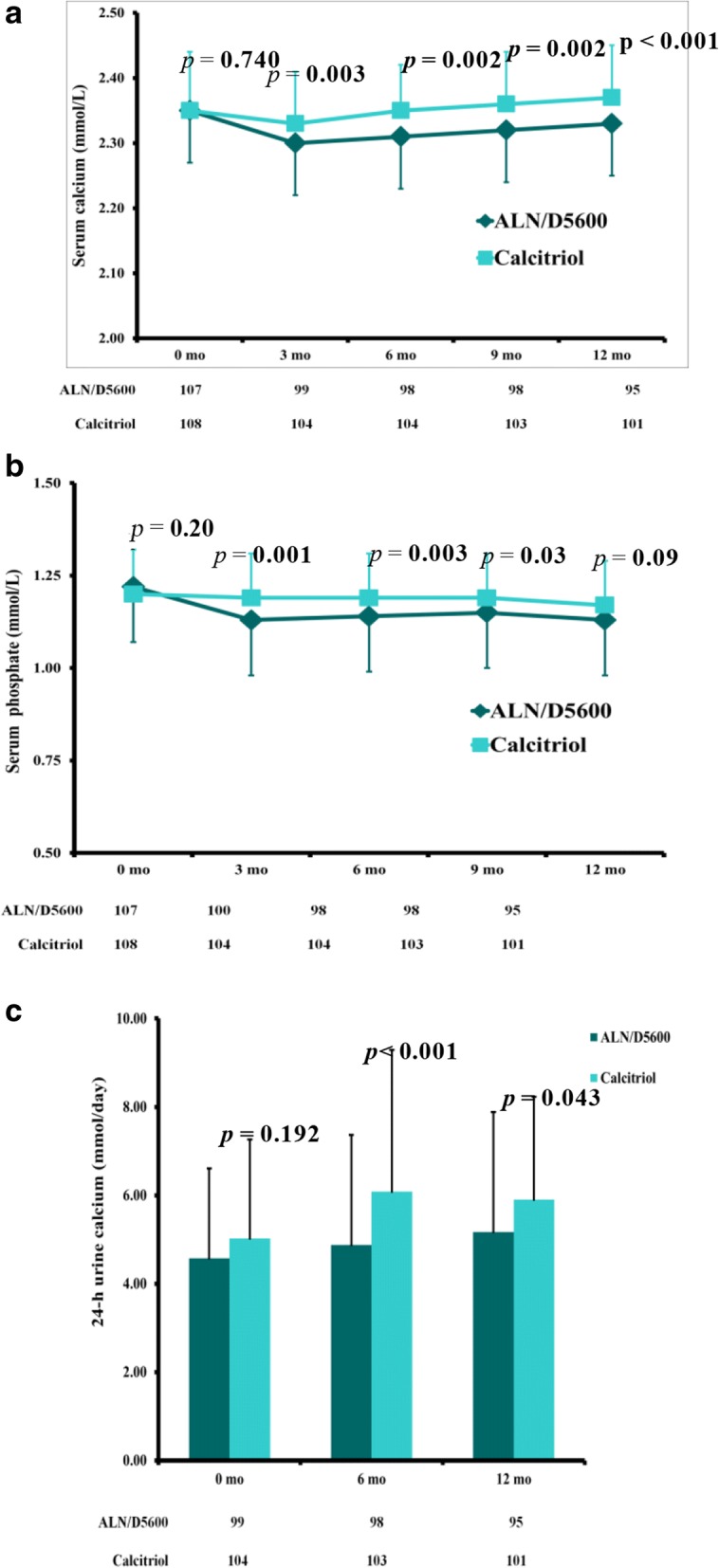
Calcium/phosphate metabolism safety through month 12 (on-treatment analysis): (**a**) mean serum calcium level; (**b**) mean serum phosphate level; (**c**) mean 24-h urine calcium level. Error bars represent the standard deviation

The frequencies of calcium-phosphate metabolism-associated events were similar between the ALN/D5600 treatment group and calcitriol treatment group, except for hypercalciuria at 6 months, with a marginal significance (9.4% vs. 18.5%, *P* = 0.05) (Table 2). In the ALN/D5600 treatment group, a significant decrease in mean serum calcium occurred at month six (− 0.04 mmol/L, *P* = 0.001 vs. baseline), but this was resolved at month 12 (*P* = 0.49 vs. baseline) Additional file 2: Table S2. Patients who received ALN/D5600 treatment had significantly lower serum calcium levels compared to those with calcitriol treatment throughout the study period (all *P-values* < 0.01, *P* < 0.001 at month 12) (Fig. 4a). Mean serum phosphate level generally suggested a similar trend. The ALN/D5600 treatment group had a significantly lower serum phosphate level than in the calcitriol treatment group at 3, 6 and 9 months (all *P*-values < 0.05), which remained similar between these two groups at 12 months *(P* = 0.09) (Fig. 4b). ALN/D5600 treatment resulted in a significantly lower 24-h urine calcium excretion compared with calcitriol treatment at months six (4.9 vs. 6.1 mmol/d) and 12 (5.2 vs. 5.9 mmol/d) (both, *P* < 0.05; Fig. 4c).

**Table 2:** Calcium-phosphate metabolism-associated AEs

*n* (%)	ALN/D5600 (*n* = 107)	Calcitriol (*n* = 108)	*P*-value
*Hypercalcemia*	0 (0.0)	1 (0.9)	0.84
*Hyperphosphatemia*	3 (2.8)	1 (0.9)	0.31
*Hypercalciuria at 6 months*	10 (9.4)	20 (18.5)	0.05
*Hypercalciuria at 12 months*	13 (12.3)	14 (13.0)	0.86

Hypercalcemia was defined as serum calcium > 2.60 mmol/L; hyperphosphatemia was defined as serum phosphate > 1.46 mmol/L with an increase of > 20% from baseline; hypercalciuria was defined as 24-h urine calcium > 7.5 mmol/L with an increase of > 25% from baseline. Data were analyzed in the APaT (all patients, as treated) population

## Discussion

Bisphosphonates and vitamin D analog are two classes of medications that are most frequently prescribed to PMO women, and both of which have been documented to have a long-term benefit in the reduction of fragility fractures [11, 12]. The combination of bisphosphonate and vitamin D analog shows a synergistic effect on skeletal and muscular systems, which is possibly due to the difference in the mode of action between these two regimens [13]. The previous randomized, controlled study conducted by the investigators also revealed that the ALN/D5600 combination was superior to calcitriol alone in PMO women with respect to BMD gain and bone turnover reduction [9]. Moreover, a subgroup analysis confirmed the difference in predictive factors for BMD response after 12-month treatment with ALN/D5600 vs. calcitriol [10]. This post hoc analysis further demonstrated that VD insufficiency/deficiency prevailed in Chinese PMO patients (approximately 90 and > 50% , respectively), and necessitated medical intervention with both antiresorptive therapy and VD supplementation. The present study confirmed that the combination of native VD with alendronate could significantly improve VD status in Chinese PMO patients with complicating VD insufficiency/deficiency. This supplementation also revealed a favorable safety profile in terms of serum 25(OH) D level and calcium-phosphate metabolism.

The long-term benefit of VD supplementation has been documented in healthy postmenopausal women with respect to bone health, muscle strength and cardiovascular risk [14–16]. A daily dose of 800–1000 IU of VD supplementation is generally recommended for Western populations [17]. The present results revealed that a weekly oral administration of 5600 IU of VD in the ALN/D5600 combination could improve VD insufficiency and deficiency in Chinese PMO women. It was also noted that following VD supplementation, Chinese PMO patients had a relatively greater proportion of patients with VD insufficiency, compared to that in the Western patient cohort (at 6 months, 47.9% vs. 8.6%) [18]. Furthermore, previous randomized controlled trials on high-dose VD supplementation revealed no additional bone health benefit [7, 19], and that increasing the VD supplementation dosage is not recommended. Therefore, additional non-medical intervention such as dietary supplementation, sun exposure and physical exercise may be needed for PMO patients with complicating VD insufficiency or deficiency.

The biochemical response of vitamin D supplementation is associated with multiple factors, including age, gender, race, body mass index, residential latitude, dietary vitamin D intake, physical exercise, season and baseline serum 25(OH) D [20]. The present results revealed that patients with low baseline vitamin D had greater serum 25(OH) D increase , confirming the efficacy of the co-administration of native vitamin D in these high-risk patients. In particular, the linear regression and LDA model suggested that the greatest change in serum VD was observed among those with the lowest baseline levels. Considering the fact that hypovitaminosis D prevails in PMO women [21], adequate supplementation with vitamin D and calcium should be given to PMO women to maintain a 25(OH) D of at least above 20 ng/mL [22]. However, there is lack of evidence that the maintenance of higher levels of 25(OH) D would benefit osteoporotic patients with respect to bone and general health.

Calcium-phosphate metabolic disorder is a major safety concern regarding the use of antiresorptive bisphosphonates and active VD analogs. Both ALN/D5600 and calcitriol generally revealed good tolerability and a similar safety profile. Calcitriol is known to be associated with a higher risk of hypercalcemia compared to other vitamin derivatives, requiring the close monitoring of serum calcium and renal function [20]. Therefore, a lower dose (0.25 μg once daily), instead of the standard dose (0.25 μg twice daily), of calcitriol is usually prescribed to Chinese PMO patients, as recommended by the Chinese guideline. The present results revealed that the use of 0.25 μg of calcitriol daily is associated with higher levels of serum calcium and 24-h urine calcium excretion, compared to ALN/D5600, throughout the treatment course, although clinically significant AEs regarding calcium/phosphate metabolism revealed no statistically significant differences, and hypercalciuria occurred slightly more frequently with calcitriol. The long-term safety of calcitriol at a higher dose, which can be uptitrated to 0.5 μg twice daily, should be evaluated in further follow-up studies.

## Conclusions

VD insufficiency/deficiency concomitantly prevailed in Chinese PMO patients, both of which required specific medical intervention for this patient population. The combination of native VD with alendronate could significantly improve the VD status of PMO patients with complicating VD insufficiency/deficiency, which remains below the safety threshold. Baseline VD status also predicted an on-treatment serum 25(OH) D increase in a significantly negative correlation. ALN/D5600 and calcitriol treatment (0.25 μg daily) revealed a similar safety profile regarding calcium-phosphate metabolism. However, calcitriol treatment was associated with a more pronounced effect on serum calcium level and urine calcium excretion. This finding necessitates the reevaluation of the calcitriol safety profile at an internationally recommended dosage higher than 0.25 μg daily in Chinese PMO patients with complicating VD insufficiency/deficiency.

## Additional files

Additional file 1: **Table S1.** Demographic and clinical characteristics of patients with VD deficiency persistent at 12 months (*n* = 4). (DOC 30 kb)

Additional file 2: **Table S2.** Calcium levels overtime in the study. (XLSX 13 kb)

## Abbreviations

BMD: Bone mineral density
BTMs: Bone turnover markers
PMO: Postmenopausal osteoporosis
VD: Vitamin D
