# Supplementary material for: Calcifediol (25-hydroxyvitamin D) improvement and calcium-phosphate metabolism of alendronate sodium/vitamin D3 combination in Chinese women with postmenopausal osteoporosis: a post hoc efficacy analysis and safety reappraisal
Source: BMC Musculoskelet Disord. 2018 Jul 3;19:210. doi: 10.1186/s12891-018-2090-y (PMC6030763; doi:10.1186/s12891-018-2090-y)
Supplement: Supplementary file 1 — Table S1. Demographic and clinical characteristics of patients with VD deficiency persistent at 12 months (n = 4). (DOC 30 kb) [file 12891_2018_2090_MOESM1_ESM.doc]

Supplementary Table 1. Demographic and clinical characteristics of patients with VD deficiency persistent at 12 months (*n*=4)

| ID | Age (year) | BMI (kg/m^2^) | Concomitant medical condition | Serum 25(OH)D level (ng/ml) | | |
| --- | --- | --- | --- | --- | --- | --- |
|  |  |  |  | 0 month | 12 months | Absolute change |
| 10300013 | 64 | 24.4 | Not significant | 9 | 19 | 10 |
| 10400017 | 59 | 28.6 | Compressive vertebral fracture | 11 | 19 | 8 |
| 10200018 | 56 | 20.4 | Osteoarthritis | 23 | 15 | -8 |
| 10100015 | 59 | 27.4 | Gallstone and cholecystectomy | 10 | 12 | 2 |
